# Comparing machine learning and artificial neural network models in psychological research: a ROC-based analysis

**DOI:** 10.3389/fpsyg.2026.1746479

**Published:** 2026-02-20

**Authors:** Marie-Luise Leitner, Martin Arendasy

**Affiliations:** Department of Psychology, University of Graz, Graz, Austria

**Keywords:** artificial neural network, decision tree, feature importance, logistic regression, machine learning, noise, overfitting, ROC (receiver operating characteristic)

## Abstract

**Introduction:**

The increasing use of data-driven methods in psychological assessment has raised the question of whether artificial neural networks provide advantages over established machine learning approaches in applied selection contexts. In particular, comparative evidence based on ROC-based evaluation using real-world psychological datasets remains limited.

**Methods:**

Using a dataset of *N* = 4,155 applicants from a university entrance examination, this study compared three traditional machine learning models—logistic regression, decision tree, and random forest—with a feedforward artificial neural network comprising a single hidden layer. All models were implemented in Python and evaluated using accuracy and receiver operating characteristic (ROC) analysis, with the area under the curve (AUC) as the primary performance metric.

**Results:**

Logistic regression achieved the highest predictive performance (accuracy = 0.973, AUC = 0.99), followed closely by the random forest model (accuracy = 0.961, AUC = 0.98). The artificial neural network reached competitive accuracy (0.933) but showed reduced discriminative ability (AUC = 0.87) and indications of overfitting. Feature importance analyses consistently identified biology, chemistry, and numerical reasoning as the most influential predictors of admission success.

**Discussion:**

The results indicate that for medium-sized, structured psychological datasets, traditional machine learning models provide more stable, interpretable, and robust performance than the evaluated shallow neural network architecture. These findings highlight the importance of model choice and inductive bias in applied psychological research and support the continued use of classical machine learning approaches in selection and assessment contexts.

## Introduction

In recent years, the increasing availability of psychological data has prompted researchers to explore advanced computational models for classification, prediction, and decision support. Machine learning (ML) and artificial neural networks (ANNs) have emerged as powerful tools in this context, offering new possibilities for identifying patterns and forecasting outcomes based on complex, multidimensional datasets (Jordan and Mitchell, 2015). In particular, their use in educational psychology has gained momentum, as researchers seek to improve the accuracy and interpretability of models used to predict academic success, diagnose learning difficulties, or inform admissions decisions (Baker and Inventado, 2014).

While neural network-based methods have received substantial attention due to their remarkable success in domains such as image recognition and natural language processing (LeCun et al., 2015), their advantages are less evident in domains characterized by relatively small sample sizes, structured tabular data, and high predictor-to-sample ratios - conditions commonly found in psychological research (Bzdok et al., 2018). In such cases, traditional machine learning algorithms like logistic regression, decision trees, and ensemble methods (e.g., random forests) have often demonstrated superior performance, both in terms of predictive accuracy and model interpretability (Shmueli, 2010; Rudin, 2019).

This study builds on this growing body of work by systematically comparing traditional ML methods and ANN architectures for a binary classification task - predicting whether individuals are “selected” or “not selected” for university admission. The dataset includes a variety of sociodemographic, academic, and cognitive variables from 4,155 participants, reflecting the kind of high-dimensional but moderately sized data typical in applied psychology.

Specifically, this paper evaluates four models: logistic regression, decision tree, random forest (as representatives of traditional ML), and a feedforward artificial neural network (ANN). Model performance is assessed using both overall accuracy and Receiver Operating Characteristic (ROC) analysis, which provides a threshold-independent measure of classification performance (Fawcett, 2006). Special attention is paid to issues of overfitting and generalization, particularly in neural network-based models, where high accuracy on training data does not necessarily translate to robust performance on test data.

Integrating ROC methodology, this research extends beyond conventional accuracy metrics and provides a robust, theory-informed framework for evaluating psychological classification models. This approach enables not only better discrimination analysis but also methodological transparency in model selection and evaluation.

The relevance of this methodological comparison extends beyond mere performance metrics. In applied settings such as methodological selection or psychological diagnosis, model interpretability, computational efficiency, and reliability under limited data conditions are essential. Addressing these practical and theoretical concerns, the present study contributes to evidence-based decision-making in psychology, while also offering methodological guidance for researchers selecting predictive models under real-world constraints.

Ultimately, this research aims to answer the following questions: How do traditional machine learning models compare to neural network-based models in terms of accuracy, generalizability, and robustness when applied to psychological datasets? What are the implications of these findings for future research and practice in psychology, education, and the social sciences?

### Introduction to receiver operating characteristic (ROC) analysis

In the evaluation of predictive models, particularly in applied psychology, medicine, and machine learning, it is crucial to assess not only the overall accuracy of a model but also its ability to distinguish between classes under varying decision thresholds. Receiver Operating Characteristic (ROC) analysis has emerged as a gold standard methodology for this purpose, offering both a conceptual and quantitative framework for evaluating classifier performance across a continuum of threshold settings (Fawcett, 2006; Metz, 1978).

**Figure 1:**
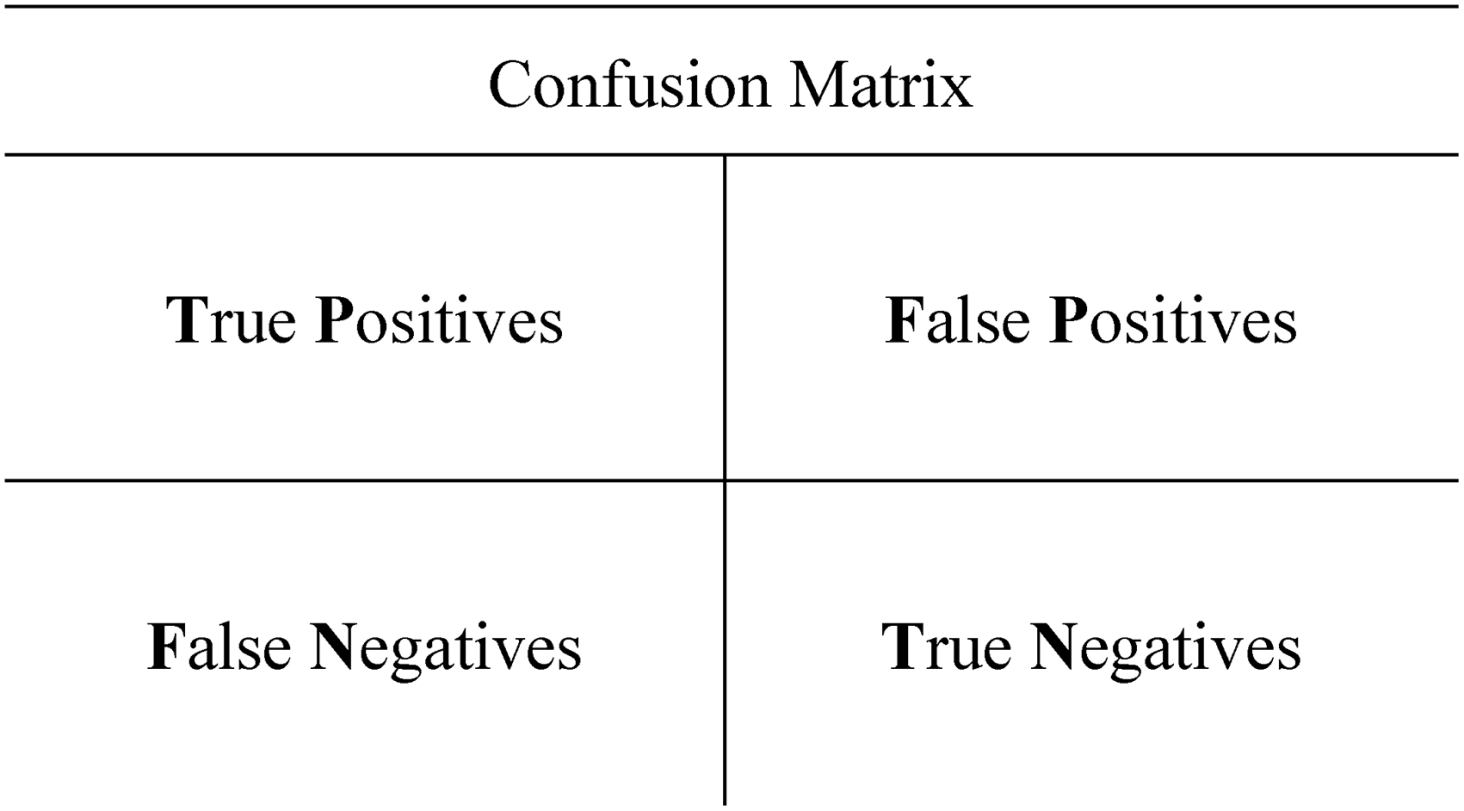
Confusion matrix (binary classification). Rows = actual class; columns = predicted class. Cells show counts [or percentages]. Each instance falls into one of four outcomes—true positive (TP), false positive (FP), false negative (FN), or true negative (TN); diagonal cells (TP, TN) are correct classifications; off-diagonals (FP, FN) are errors.

The ROC framework is built on the confusion matrix (Figure 1):

The *true positive* rate, which is also named *sensitivity, hit rate* or *recall*, is calculated by the following expression (Fawcett, 2006):The performance measures used in ROC analysis and model evaluation are formally defined in Equations 1–13.


$$\mathrm{TPR}\;(\text{True Positive Rate})=\frac{\text{Sensitivity}}{\text{Recall}}=\frac{\mathrm{TP}}{\mathrm{TP}+\mathrm{FN}}$$ (1)

The *false positive* rate, also referred to as a *false alarm,* is expressed as


$$\mathrm{FPR}\;(\text{False Positive Rate})=\text{False Alarm}=\frac{\mathrm{FP}}{\mathrm{TN}+\mathrm{FP}}$$ (2)

The true negative rate is denoted as specificity, while the false negative rate corresponds to the false omission rate or the complement of sensitivity.


$$\mathrm{TNR}\;(\text{True Negative Rate})=\text{Specificity}=\frac{\mathrm{TN}}{\mathrm{TN}+\mathrm{FP}}$$ (3)

$$\mathrm{FNR}\;(\text{False Negative Rate})=\frac{\mathrm{FN}}{\mathrm{FN}+\mathrm{TP}}$$ (4)

Stated differently, sensitivity and specificity quantify two distinct aspects of classification accuracy with respect to a binary outcome—such as passing or missing an entrance test. Sensitivity refers to the proportion of actual positive cases (e.g., applicants who would genuinely pass) that are correctly identified by the test, whereas specificity refers to the proportion of actual negative cases (e.g., those who would not pass) that are correctly classified as such. These metrics can be applied to any binary classification problem, provided that the outcome categories are clearly operationalized in the process of calculating and interpreting sensitivity and specificity values (Metz, 1978).

In addition, three other terms used in ROC analysis represent conditions involving negative cases and incorrectly identified positive cases (Metz, 1978):


$$\text{Precision}=\frac{\mathrm{TP}}{\mathrm{TP}+\mathrm{FP}}$$ (5)

$$\text{Accuracy}=\frac{\mathrm{TP}+\mathrm{TN}}{\mathrm{TP}+\mathrm{FP}+\mathrm{FN}+\mathrm{TN}}$$ (6)

**Figure 2:**
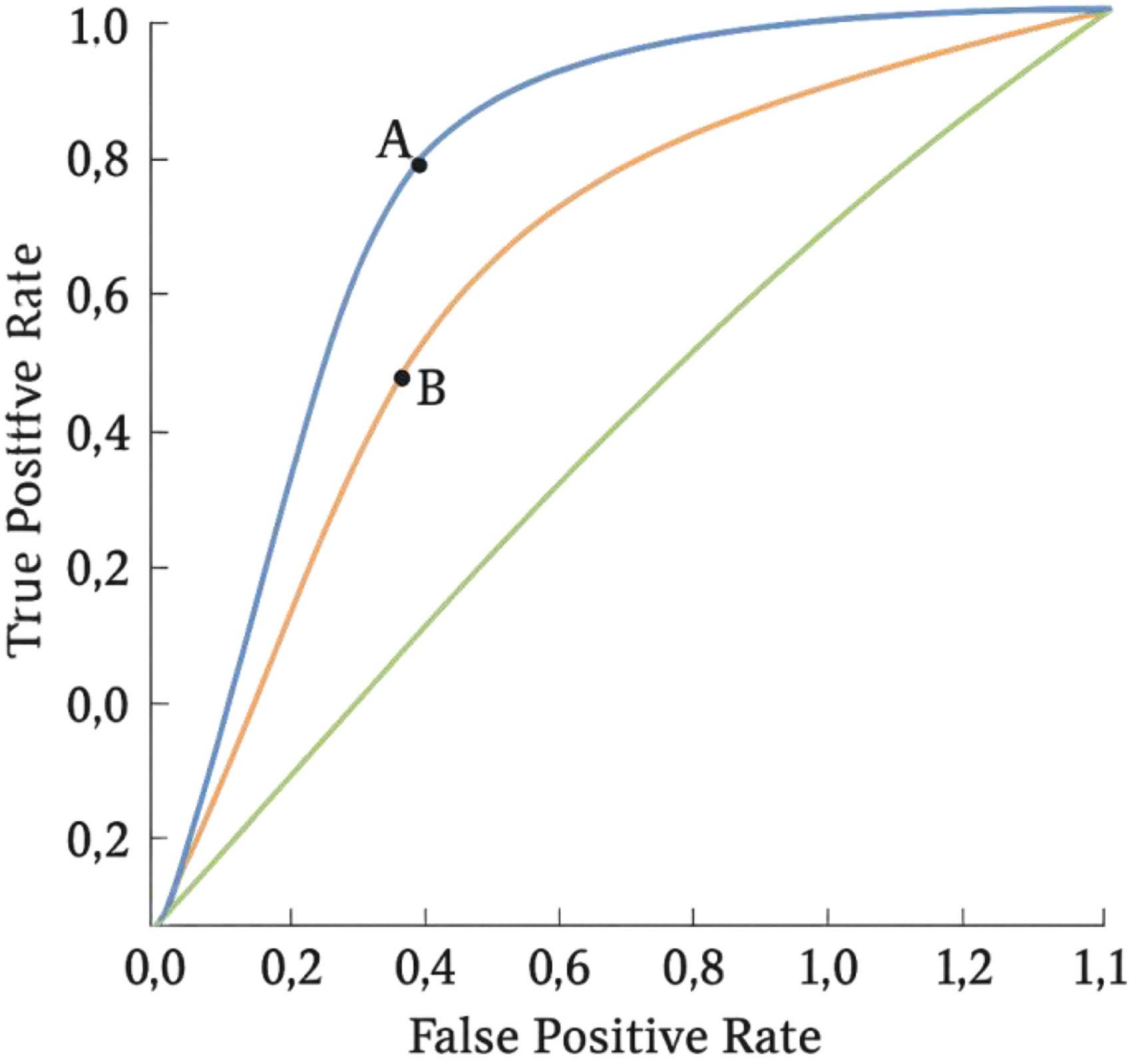
ROC curves with threshold points. ROC curves for three classifiers plotting true positive rate (TPR) against false positive rate (FPR) across decision thresholds. The blue curve shows the strongest discrimination, followed by orange and green (larger AUC implies better performance). Points A and B mark specific thresholds: A prioritizes sensitivity (higher TPR, higher FPR), while B is more conservative (lower FPR, lower TPR). Curves closer to the top-left corner indicate superior performance; a diagonal chance line would reflect random classification. The false positive rate on the *x*-axis ranges from 0 to 1.

Traditional accuracy metrics often obscure critical nuances of classification performance, particularly when the base rates of the outcome classes are imbalanced. As Metz (1978, 1979) illustrated using diagnostic screening examples, a classifier may achieve high nominal accuracy by simply favouring the majority class yet fail catastrophically in identifying the minority class of interest. This limitation necessitates a more refined metric that captures the trade-off between true positive and false positive rates—a gap that ROC analysis is designed to address (Figure 2).

In psychology, ROC curves are particularly valuable for evaluating tests and classification models where different types of errors—false positives versus false negatives—carry different theoretical and practical consequences (Swets, 1988; Streiner and Cairney, 2007).

From a methodological standpoint, ROC analysis enables three core applications:

1. Threshold selection: By analyzing the ROC curve shape and slope at various points, researchers can determine the optimal cut-off value based on the cost–benefit trade-offs of false positives and false negatives (Westin et al., 2001).
2. Comparing classifiers: AUC values allow for model comparison regardless of scale or unit. However, overlapping or crossing ROC curves necessitate more nuanced statistics, such as the partial AUC or resampling methods (Faraggi and Reiser, 2002). The statistical interpretation of the area under the ROC curve (AUC) was formally established by Hanley and McNeil (1982), providing a foundation for subsequent ROC-based model comparisons.
3. Discrimination capacity: The ROC curve facilitates understanding of a model’s capacity to distinguish between groups—an essential feature in psychological test construction, where latent traits must be inferred from observable indicators.

While ROC analysis is valuable for assessing discriminative ability, it does not account for the calibration of predicted probabilities and may be less informative in highly imbalanced datasets. In such cases, precision–recall curves may offer a useful complement (Saito and Rehmsmeier, 2015).

The current study employs ROC analysis as a central evaluation tool to compare four classification algorithms: logistic regression, decision tree, random forest, and single-layer neural network. For each model, the ROC curve and its associated AUC are calculated based on out-of-sample test predictions. In addition, the analysis encompasses ROC-based threshold optimization, comparative evaluation of area under the curve (AUC), and the examination of classifier bias as represented within the ROC space.

### Introduction to logistic regression

Logistic regression is one of the most widely used and foundational models in both psychological research and statistical classification. As a generalized linear model (GLM), it provides a robust framework for estimating the probability of a binary outcome based on one or more predictor variables (Hosmer et al., 2013; Agresti, 2013). Its enduring appeal in psychology stems from its interpretability, statistical rigor, and capacity for inference, making it suitable for both hypothesis testing and predictive modeling (Menard, 2002; Pampel, 2000).

The logistic regression model is governed by a specific mathematical function, which can be described as follows:


$$\hat{y}=b_{1}.x_{1}+b_{2}.x_{2}+\ldots +b_{k}.x_{k}+a$$ (7)

Expressed in words, the predicted probability of a binary outcome is calculated by taking the exponential of a linear combination of predictor variables ( $x_{1},x_{2},\ldots ,x_{k})$ multiplied by their corresponding coefficients ( $b_{1},b_{2},\ldots ,b_{k}),$ and adding a constant term (a). This linear combination is then transformed using the logistic or sigmoid function, which projects the outcome into a binary value between 0 and 1. The result value represents the estimated probability of the binary outcome, indicating the likelihood of belonging to a particular category or class.

Given a linear combination:


$$z=\beta_{0}+\beta_{1}x_{1}+\beta_{2}x_{2}+\ldots +\beta_{k}x_{k}$$ (8)

…the logistic function transforms it into the probability:


$$P(Y=1X)=\frac{1}{1+e^{-z}}=\frac{1}{1+e^{-(\beta_{0}+\beta_{1}x_{1}+\beta_{2}x_{2}+\ldots +\beta_{k}x_{k})}}$$ (9)

This transformation maps the linear predictor onto a probability scale from 0 to 1, representing the estimated likelihood of an observation belonging to a particular category or class (Long and Freese, 2014).

In applied psychology, these assumptions are generally satisfied when working with moderate to large datasets and well-curated instruments. However, violations—particularly of multicollinearity and sample size may bias estimates and reduce generalizability (Babyak, 2004).

In the present study, logistic regression serves as the benchmark for evaluating more complex models, including decision trees, random forests, and neural networks. Its relatively simple architecture allows for high interpretability, and its well-established statistical underpinnings facilitate construct validation and inference, which are often required in psychology but less straightforward with black-box models (Breiman, 2001a, 2001b; Yarkoni and Westfall, 2017).

### Introduction to decision trees

Decision trees are a class of supervised learning algorithms used for classification and regression tasks. They function by recursively partitioning the feature space into subsets based on input variables, creating a tree-like structure composed of decision nodes and terminal leaves (Breiman et al., 1984). At each node, the algorithm selects the feature and corresponding split point that optimally separates the data according to a predefined impurity criterion, such as Gini impurity, information gain (based on entropy), or classification error.

As non-parametric models, decision trees do not assume any specific distribution of the input data, which makes them particularly attractive in applied psychological and educational research where assumptions of linearity and normality are often violated. The model construction typically follows a greedy, top-down approach known as recursive binary splitting, aiming to produce subsets that are as homogeneous as possible with respect to the target variable (Breiman et al., 1984).

Mathematically, for a given node *t*, the impurity *I(t)* can be measured using Gini impurity:


$$I(t)=1-\sum_{i=1}^{C}p_{i}^{2}$$ (10)

Despite their simplicity and interpretability, decision trees are highly sensitive to overfitting, particularly when grown to full depth without regularization constraints. In such cases, they may model random noise in the training data as if it were meaningful structure, thereby reducing their generalizability to unseen cases (Quinlan, 1997; Balcan and Sharma, 2024). To mitigate this risk, pruning strategies—such as pre-pruning and post-pruning—are commonly employed, alongside the specification of minimum sample thresholds per node, in order to reduce model complexity and enhance robustness (Bramer, 2002; Ahmed et al., 2018).

The interpretability of decision trees, conveyed through easily understandable decision rules, makes them particularly valuable in domains where transparency is essential, such as clinical diagnostics, personnel selection, or university admissions (Blockeel et al., 2023; Agarwal et al., 2022a, 2022b). However, due to their methodological limitations—namely high variance and sensitivity to minor perturbations in the data—caution is warranted when applying them to high-stakes decisions. These challenges often motivate the use of ensemble techniques, such as random forests or gradient-boosted trees, which aggregate multiple decision trees to produce more stable and accurate predictions (Breiman, 2001a, 2001b; Balcan and Sharma, 2024).

### Introduction to random forests

Random Forests represent a powerful and widely used ensemble learning method in supervised machine learning, particularly suitable for both classification and regression tasks (Breiman, 2001a, 2001b). As an extension of decision tree models, Random Forests aim to overcome the high variance and overfitting tendencies of individual trees by aggregating predictions from multiple decision trees built on random subsets of data and features.

At the core of the Random Forest algorithm lies the principle of bootstrap aggregating, or *bagging* (Breiman, 2001a, 2001b). This process involves generating multiple bootstrap samples from the training data by sampling with replacement. For each sample, a separate decision tree is constructed. At every node split during tree construction, a random subset of features (rather than all features) is evaluated to determine the optimal split. This dual randomization - in both sample selection and feature selection—introduces model diversity and reduces the correlation between individual trees, thereby improving the generalization performance of the ensemble (Hastie et al., 2009).

Mathematically, the final prediction of a Random Forest is the aggregated outcome of all individual trees. For classification tasks, this typically involves a majority vote across the trees:


$$\hat{y}=\text{mode}\{T_{1}(x),T_{2}(x),\ldots ,T_{n}(x)\}$$ (11)

For regression, the prediction is the mean of the outputs:


$$\hat{y}=\frac{1}{n}\sum_{i=1}^{n}T_{i}(x)$$ (12)

where $\;T_{i}(x)\;\text{denotes the prediction of the}\;i-\mathrm{th}\;\text{tree},\text{and}\;T\;$ is the total number of trees in the forest.

Random Forests offer several advantages, particularly in applied research contexts. They handle large, high-dimensional datasets efficiently, are robust to outliers and noise, and can deal with missing values without the need for imputation (Ayyadevara, 2018). Moreover, they provide internal metrics such as feature importance scores and out-of-bag (OOB) error estimates, which allow for an efficient and unbiased estimation of generalization error without requiring a separate validation set (Liaw and Wiener, 2002).

In psychological methodology, Random Forests are especially valuable due to their non-parametric nature, eliminating the need to satisfy stringent assumptions such as linearity, normality, or homoscedasticity. For instance, Fife and D’Onofrio (2023) show that Random Forests outperform traditional regression models under conditions of nonlinear effects and interaction among predictors. In studies of reading ability, Matsuki et al. (2016) demonstrate that Random Forests better manage overfitting and multicollinearity in datasets with many highly correlated predictors.

Despite their robustness and flexibility, Random Forests are not without limitations. Their ensemble-based architecture, which aggregates predictions from a large number of decorrelated decision trees, makes it difficult to trace how individual input variables influence a specific classification outcome. This lack of transparency limits their interpretability, particularly in comparison to models such as logistic regression, which offer coefficient-based inference, or single decision trees, which provide rule-based explanations (Breiman, 2001a, 2001b; Fife and D’Onofrio, 2023; Matsuki et al., 2016). In contexts such as psychological assessment, educational placement, or admissions testing—where interpretability and justification of decisions are critical—this limitation poses a significant challenge.

Moreover, Random Forest performance may degrade in the presence of severely imbalanced datasets, as the algorithm tends to favour the majority class. This issue is particularly relevant in high-stakes classification tasks, where the minority class often represents the group of primary interest (e.g., students at risk of failing an entrance test). Without corrective measures, such as resampling techniques, class weighting, or cost-sensitive learning, predictive performance for the minority class may be substantially compromised (Chen et al., 2004; Branco et al., 2016).

Overall, Random Forests present a compelling methodological choice when prediction accuracy, noise resilience, and variable importance estimation are prioritized over model transparency. In the context of this dissertation, they are employed as a comparative benchmark against other models (e.g., logistic regression, support vector machines, neural networks) to evaluate classification accuracy, AUC performance, and resistance to overfitting.

### Introduction to artificial neural network (ANN)

Artificial neural networks (ANNs) are computational models inspired by the architecture and functioning of the human brain. Originally developed to emulate biological neural systems, ANNs are particularly well suited for solving complex, non-linear problems that are intractable for traditional statistical approaches (Haykin, 2009). Their structure consists of interconnected processing units (neurons) organized in layers, allowing them to learn data representations through iterative training processes.

The typical ANN comprises an input layer, one or more hidden layers, and an output layer. Each neuron in a layer is connected to neurons in the subsequent layer via weighted connections. During the forward pass, neurons compute weighted sums of their inputs and apply an activation function (e.g., sigmoid, ReLU, or softmax) to introduce non-linearity into the model. The model’s predictive capacity is refined through backpropagation, a learning algorithm that minimizes a loss function by adjusting weights based on the gradient descent principle (Haykin, 2009).

Mathematically, for a neuron $j,\text{the activation}\;a_{j}\;i$ s given by:


$$a_{j}=\phi (\sum_{i=1}^{n}w_{\mathrm{ij}}x_{i}+b_{j})$$ (13)

$$\begin{array}{l} \text{where}\;x_{i}\;\mathrm{are}\;\text{the input values},w_{\mathrm{ij}}\;\mathrm{are}\;\text{the corresponding weights},\\ b_{j}\;\text{is the bias term},\text{and}\;\phi (\cdot )\;\text{is the activation function}. \end{array}$$

A central strength of artificial neural networks (ANNs) is their theoretical capacity to approximate any continuous function to an arbitrary degree of accuracy, a property known as universal approximation: under certain conditions, a neural network with just one hidden layer can approximate any continuous function on compact subsets of ℝⁿ, given sufficient neurons (Hornik, 1991). This theoretical property makes ANNs especially attractive for modeling psychological data, which often exhibit non-linear interactions and latent patterns.

There are three main types of ANN training methods: supervised learning, in which input–output pairs guide weight adjustments; unsupervised learning, where the network identifies structure in unlabelled data; and self-supervised or fixed-weight networks, used in constrained optimization scenarios (Fausett, 1994).

In psychological research, ANNs are increasingly utilized to detect complex relationships in behavioural, cognitive, and neuropsychological data. Their ability to model non-linear associations, handle high-dimensional inputs, and learn from noise makes them a valuable methodological tool—though they often lack interpretability, which can be a limitation in theory-driven research.

In this study, an artificial neural network (ANN) was implemented to classify admission outcomes based on psychometric features. Performance is evaluated against classical and modern machine learning techniques (e.g., logistic regression, decision trees, support vector machines, and random forests) using metrics such as accuracy, AUC, and ROC analysis.

### Aim and significance of the study

The primary aim of this study is to systematically evaluate and compare the predictive performance of traditional machine learning models (logistic regression, decision tree, random forest) and modern neural network-based model approaches (i.e., a feedforward artificial neural network) in classifying outcomes within a psychological admissions dataset. Using a real-world dataset of *N* = 4,155 applicants, the study investigates the utility, accuracy, and robustness of these models in identifying individuals who are likely to succeed in an entrance examination based on demographic, academic, and cognitive features.

This research is significant in both theoretical and practical terms. Methodologically, it contributes to the ongoing discussion about the appropriateness of neural network-based models in psychological context, where sample sizes are typically smaller than in industrial machine learning applications. Practically, the findings have implications for optimizing selection procedures in applied psychological assessment by identifying the most efficient and interpretable predictive models for use in personnel selection, educational screening, and diagnostic processes.

### Research questions

This study systematically examines the comparative utility of traditional and modern machine learning approaches in the classification of psychological data. The investigation is guided by the following research questions:

- To what extent does the predictive performance of neural network-based models differ from that of traditional statistical methods when applied to psychological classification problems?
- Among the selected classification algorithms: logistic regression, decision trees, random forests, and artificial neural networks—which demonstrates the highest level of predictive accuracy in the given psychological dataset?
- Given the available dataset (N > 4,000), neural network-based models exhibit signs of overfitting, and what are the methodological and practical implications of such behaviour for their application in psychological research contexts?
- What are the respective methodological advantages and limitations of traditional classification techniques and neural network-based models, particularly when applied to small or moderately sized psychological datasets?

## Methods

### Participants

The dataset consisted of *N* = 4,155 applicants to a university entrance examination in health sciences. The data were collected retrospectively and included a wide range of sociodemographic, academic, and cognitive features. As the dataset was fully anonymized and archival in nature, no direct interaction with human participants took place, and ethical approval was not required. Nonetheless, data were handled in compliance with data protection regulations.

After initial data cleaning, including the removal of outliers, missing values, and implausible or falsified entries (e.g., incorrect age specifications), the dataset was reduced from *N* = 4,177 to *N* = 4,155 applicants. The final sample comprised 2,447 females and 1,708 males, ranging in age from 18 to 38 years. Regarding nationality, 2,756 participants were Austrian citizens, 1,294 originated from other European Union (EU) countries, and 105 from non-EU countries. Participants represented a broad range of secondary school backgrounds, including Gymnasium, Realgymnasium, Oberstufenrealgymnasium, Naturwissenschaftliches Gymnasium, Humanistisches Gymnasium, Neusprachliches Gymnasium, foreign school-leaving certificates, Handelsakademie, technical and vocational colleges, schools of business administration, and other school types. Academic indicators included subject-specific knowledge in biology, chemistry, physics, and mathematics. Cognitive ability measures covered figural analogies (*fz_score*), number series (*zf_score*), memory performance (*gm_score*), and mathematical thinking (*md_score*). Text processing competence (*tv_score*) was also included. The binary dependent variable was admission outcome (*sel*; 0 = not selected, 1 = selected). To assess possible redundancy among predictors, intercorrelation analyses were conducted. The shared variance (*R*^2^) among predictors was consistently low, with all pairwise correlations remaining below r = 0.70. In addition, variance inflation factor (VIF) values were below the conventional threshold of 5, and tolerance statistics exceeded 0.20, indicating that multicollinearity was not a concern. Taken together, these results suggest that the predictors could be considered sufficiently independent for the purposes of model estimation (Dangeti, 2017; Hair et al., 2010).

### Measures

*Sociodemographic variables*. Participants reported gender (female, male), age (18–38 years), nationality (Austria, EU, non-EU), and type of secondary school attended (e.g., Gymnasium, Realgymnasium, Oberstufenrealgymnasium, Naturwissenschaftliches Gymnasium, Humanistisches Gymnasium, Neusprachliches Gymnasium, foreign school-leaving certificate, Handelsakademie, technical and vocational college, school of business administration, or other).

*Cognitive ability measures*. Cognitive performance was assessed using subtests that captured (a) figural reasoning (*fz_score*), (b) number series (*zf_score*), (c) memory performance (*gm_score*), and (d) mathematical thinking (*md_score*). These indicators reflect core dimensions of general cognitive ability relevant to academic success.

*Academic knowledge measures*. Domain-specific knowledge was measured through subject-based test scores in biology (*bi_score*), chemistry (*ch_score*), physics (*ph_score*), and mathematics (*ma_score*).

*Text processing competence*. In addition, a standardized task assessing text processing skills (*tv_score*) was included as an indicator of verbal-academic competence.

*Outcome variable*. The dependent variable was admission outcome (*sel*), coded dichotomously as 0 = not selected and 1 = selected.

### Procedure

The research followed a quantitative, data-driven modelling framework. All models were implemented in Python using open-source libraries such as Scikit-learn, Keras, and TensorFlow. The dataset was split into training and testing sets using an 80/20 ratio, with stratified sampling applied to preserve the distribution of the outcome variable. Each model was trained and tested on the same data split to ensure direct comparability of performance metrics.

Responses were automatically recorded and scored using standardized algorithms. Data integrity was ensured through immediate plausibility checks at the point of entry. Following data collection, all records were anonymized so that no personal identifiers were retained. The binary admission outcome was determined based on official university admission criteria and subsequently linked to each participant’s test record.

### Data preprocessing

All categorical variables, including those representing nationality and type of secondary school attended, were transformed using one-hot encoding to facilitate their inclusion in the machine learning models. Numerical variables were standardized to have a mean of zero and a standard deviation of one in order to ensure comparability across features and to support the convergence of gradient-based algorithms. The dataset contained only minimal missing data, which were addressed through case-wise deletion. An analysis of class distribution revealed no substantial imbalance between admitted and non-admitted applicants (51.7% selected vs. 48.3% not selected), rendering the use of resampling techniques or class weighting unnecessary. The final dataset therefore exhibited a near-balanced class distribution, which does not constitute a substantial class imbalance according to common conventions in classification research (e.g., Fawcett, 2006). Accordingly, accuracy and ROC-based metrics were considered appropriate evaluation measures.

### Model description

Four models were implemented and compared:

- Logistic regression (LR)

A regularized logistic regression model was used as a baseline. It assumes linear relationships between predictors and the log-odds of the outcome.

II Decision tree (DT)

A Gini impurity-based classification tree was grown without pruning to evaluate model instability and overfitting tendencies.

III Random forest (RF)

An ensemble of 100 decision trees was constructed using bootstrap aggregation and random feature sampling to reduce variance and increase robustness.

IV Artificial neural network – (ANN)

The neural network implemented in the present study was a feedforward artificial neural network with a single hidden layer. This shallow architecture reflects a commonly used neural network design in applied psychological research and was selected to represent typical practical implementations under real-world conditions.

A feedforward neural network comprising a single hidden layer with 32 units and ReLU activation was implemented. The output layer consisted of a single neuron with a sigmoid activation function, suitable for binary classification. The model was trained using binary cross-entropy loss and optimized with the Adam algorithm.

All models used identical inputs and were evaluated on the same test split for comparability.

To ensure a fair and comparable evaluation across model classes, all models were implemented using commonly recommended default or conservative hyperparameter settings. No extensive hyperparameter optimization was performed for any model class. This decision was motivated by the primary aim of the study, which was to compare model robustness, generalization behaviour, and interpretability under typical applied conditions rather than to maximize predictive performance through fine-tuning.

### Evaluation metrics

To assess and compare the performance of the classification models, a set of complementary performance metrics was employed:

- Accuracy, defined as the proportion of correctly classified instances, served as a baseline measure of overall predictive performance.
- Area under the receiver operating characteristic curve (AUC-ROC) was used to quantify the model’s ability to discriminate between admitted and non-admitted applicants across all possible classification thresholds.
- Receiver operating characteristic (ROC) curves were plotted to visualize the trade-off between the true positive rate and false positive rate at varying threshold levels.
- Feature importance scores, calculated for tree-based models (e.g., decision trees and random forests), were used to identify the most influential predictor variables in the classification process.

These metrics enabled both threshold-independent and threshold-dependent comparisons between models.

### Noise as a methodological factor

In this study, noise, defined as random variability not systematically associated with the true outcome, was explicitly treated as a methodological concern. Consistent with the conceptualization by Kahneman et al. (2021), noise was understood as unwanted variability that can obscure signal and compromise model reliability. Its presence and influence were examined both qualitatively, through observed differences in model behaviour, and quantitatively, via fluctuations in predictive performance across training iterations.

Among the models evaluated, decision trees demonstrated pronounced sensitivity to noise, with considerable variability in classification outcomes observed across repeated training runs. This instability aligns with prior findings suggesting that decision trees, particularly when grown to full depth, tend to overfit due to their reliance on greedy, axis-aligned splits and their responsiveness to small perturbations in the training data (Quinlan, 1996; Rokach and Maimon, 2008). Similarly, artificial neural networks exhibited a tendency to memorize both meaningful patterns and irrelevant fluctuations in the data. Overfitting was most evident in the absence of regularization techniques such as dropout or early stopping (Zhang et al., 2017). In contrast, random forests displayed greater robustness to noise. Their ensemble-based architecture, which aggregates predictions across multiple decorrelated trees, effectively reduced variance and mitigated overfitting, thereby enhancing model stability (Breiman, 2001a, 2001b; Biau and Scornet, 2016).

Although no artificial noise was injected into the dataset, variability in model performance across repeated runs was interpreted as reflecting algorithmic instability rather than noise inherent in the data. To further strengthen this perspective, the treatment of noise was embedded into the overall evaluation framework. This ensured that performance differences were not only attributed to sampling variability but also interpreted considering each model’s structural sensitivity to random perturbations. By framing noise explicitly as a methodological dimension, the study underscores its central role in evaluating the robustness and generalizability of predictive models in psychological research.

Variability in model performance across repeated training runs may arise from several algorithmic sources, including random weight initialization, stochastic optimization procedures, and sensitivity to hyperparameter choices. In the present study, such variability is therefore interpreted as an indicator of algorithmic instability rather than as direct evidence of noise inherent in the data.

Accordingly, the term “noise” is used here in a broader methodological sense to denote unwanted variability in model behaviour, rather than explicitly introduced data perturbations.

## Results

This section reports the predictive performance of four machine learning models, each trained to classify success in a university entrance examination. All models were trained on identical feature sets and evaluated using consistent training/test splits to ensure comparability. Model performance was assessed using overall classification accuracy and, more importantly, receiver operating characteristic (ROC) analysis and the area under the ROC curve (AUC). The AUC metric, as a threshold-independent measure of discrimination, is particularly informative in evaluating model generalizability across varying decision boundaries (Fawcett, 2006). Presenting accuracy together with AUC ensures a balanced view of threshold-dependent and threshold-independent performance across models.

### Logistic regression

The logistic regression model achieved the highest classification performance, with a test accuracy of 0.973 and an AUC of 0.99. ROC analysis revealed excellent sensitivity and specificity across a wide range of threshold values. These results suggest not only high accuracy in predicting exam success but also robust generalization, making logistic regression both statistically reliable and practically interpretable.

### Decision tree

The decision tree classifier yielded a test accuracy of 0.926 and an AUC of 0.80. While the overall accuracy indicates a reasonable level of predictive performance, the lower AUC suggests that the model’s discriminative capacity is more sensitive to threshold settings. This limitation reflects the well-documented tendency of single-tree models to overfit and produce less stable decision boundaries. Post-pruning the tree (e.g., constraining maximum depth to 5) led to a marginal increase in accuracy (0.930) but no substantial improvement in AUC, indicating that pruning alone may not sufficiently enhance generalizability. The most influential features identified were biology (*bi_score* = 0.085), numerical reasoning (*zf_score* = 0.039), and memory performance (*gm_score* = 0.034).

### Random forest

The random forest classifier achieved a test accuracy of 0.961 and an AUC of 0.98. This strong performance reflects the ensemble model’s ability to aggregate across multiple decorrelated trees, thereby reducing variance and enhancing robustness to overfitting. The ROC curve demonstrated excellent class separation, confirming the model’s ability to generalize effectively. Feature-importance analysis identified prior achievement in biology (*bi_score* = 0.176), chemistry (*ch_score* = 0.132), and numerical reasoning (*zf_score* = 0.103) as the most influential predictors of exam success.

### Artificial neural network

The single-layer artificial neural network reached a test accuracy of 0.933 and an AUC of 0.87. ROC analysis showed a noticeably lower AUC compared to tree-based models, especially at training epochs 21, 42, 46, and 50, where overfitting became evident. These findings indicate that, while the ANN achieved competitive accuracy, its ability to generalize across decision thresholds was limited. The results highlight the need for regularization and careful tuning when applying neural networks to moderately sized, multivariable datasets.

### Comparative model summary

Table 1 shows accuracy of machine learning and neural network-based model.

**Table 1:** Accuracy of machine learning and neural network-based model.

Model	Accuracy (Machine learning)	Accuracy (Neural network)
Logistic regression	0.973	-
Random forest	0.961	-
Decision tree	0.926	-
Neural network	-	0.933

### Comparative feature selection

Table 2 shows comparative feature importance for decision tree and random forest models.

**Table 2:** Comparative feature importance for decision tree and random forest models.

Model	Most important feature	Second most important feature	Third most important
Random forest	Biology (*bi_score* = 0.176)	Chemistry (*ch_score* = 0.132)	Number series (*zf_score* = 0.103)
Decision tree	Biology (*bi_score* = 0.085)	Number series (*zf_score* = 0.039)	Memory performance (*gm_score* = 0.034)

### ROC-based evaluation

The ROC curves collectively demonstrate that logistic regression and random forest models yield the most reliable performance across thresholds, with ROC curves closely approaching the upper-left corner of the ROC space. These models are thus especially suitable in applied psychological contexts where decision thresholds may shift (e.g., when prioritizing false positives over false negatives in screening).

## Discussion

The present study compared the predictive performance of traditional machine learning models and neural network-based model approaches for the classification of applicants in a psychological university entrance test on a real-world dataset. Using a dataset of *N* = 4,155 cases and identical training/test splits, four classification models were evaluated with particular attention to predictive accuracy, ROC analysis, and generalization ability. The findings offer several important insights for model selection and methodological decision-making in psychological research and applied classification tasks.

Traditional models—specifically logistic regression and random forest—demonstrated the highest overall classification performance. Logistic regression achieved the best results, with an accuracy of 0.973 and a near-perfect area under the ROC curve (AUC), confirming its strong discriminatory power across classification thresholds. This aligns with its long-standing reputation in the literature as a robust, interpretable model particularly well suited for binary classification tasks in psychological contexts. Similarly, the random forest algorithm achieved high accuracy (0.961) and exhibited excellent generalization performance, with ROC curves indicating stable separation between classes. Feature importance analyses identified subject-specific competencies biology (*bi_score* = 0.176), chemistry (*ch_score* = 0.132), and numerical reasoning (number series) (*zf_score* = 0.103) as key predictors—offering both practical relevance and theoretical alignment with classical intelligence models.

### Interpretability and scope of feature importance analyses

Differences in feature importance across models do not indicate contradictory findings but rather reflect model-specific inductive biases. Feature importance measures derived from decision trees and random forests are inherently dependent on the model structure, splitting criteria, and interaction effects among predictors. While decision tree importance values are highly sensitive to individual splits and therefore less stable, random forest importance represents an aggregated, global estimate across multiple decorrelated trees and can thus be considered more robust at the model level.

Model-agnostic explanation techniques such as LIME (Ribeiro et al., 2016) provide local, instance-level explanations of individual predictions and serve a complementary purpose. By contrast, the present study focused on global model behaviour and comparative robustness rather than on post-hoc explanations of individual cases.

Accordingly, feature importance values in this study should be interpreted as model-internal relevance indicators serving comparative and descriptive purposes, rather than as causal estimates.

The decision tree model yielded the lowest overall predictive performance among the models evaluated. This outcome may be attributed to the limitations of single-tree structures when applied to multivariable, structured datasets. Unlike random forests, which aggregate predictions from multiple trees and thereby capture more complex feature interactions, individual decision trees are less capable of handling multidimensional patterns within the data. The structural simplicity of the decision tree model, while advantageous in terms of interpretability, may therefore contribute to its reduced classification accuracy.

Despite its lower overall performance, the decision tree’s feature selection results remain of interest. The model identified biology (*bi_score* = 0.085), numerical reasoning (*zf_score* = 0.039), and memory performance (*gm_score* = 0.034) as the most influential predictors. These findings may offer valuable insights for researchers interested in domain-specific item analysis or targeted test development. Notably, the feature selection outcomes of both the decision tree and random forest models converge in highlighting the importance of subject-specific knowledge (e.g., biology and chemistry) alongside domain-general cognitive abilities (e.g., numerical reasoning and memory). This suggests that these variables are particularly relevant for the accurate classification of success in the university entrance examination.

In contrast, the neural network-based model showed notably lower performance. Although the single-layer network reached an accuracy of 0.933, closer inspection of the training process and ROC curves revealed significant overfitting, with performance degradation beginning at early training epochs. The reduced AUC values indicated poor discrimination across thresholds. These patterns highlight the tendency of feedforward neural networks to memorize training data in smaller, structured datasets without appropriate regularization. The results also emphasize the challenges of applying neural network-based models to moderately sized psychological datasets, where generalization can be limited without careful regularization and architecture choices tailored to tabular data.

A theoretical explanation for this pattern can be derived from the concept of architectural inductive bias. Classical models such as logistic regression and tree-based methods impose strong, task-relevant inductive biases that align well with structured tabular data. Linear models encode additive and monotonic relationships, whereas decision trees and random forests exploit axis-aligned splits and hierarchical feature interactions. Feedforward neural networks, by contrast, rely on comparatively weak and generic inductive biases that assume smooth function approximation rather than explicitly leveraging the structural properties of tabular data. Recent research has demonstrated that this mismatch systematically disadvantages neural networks on tabular datasets, even at moderate sample sizes, whereas classical models often outperform neural network-based architectures under these conditions (Grinsztajn et al., 2022; Shwartz-Ziv and Armon, 2022; Borisov et al., 2023).

Methodologically, the findings reaffirm that simpler, traditional models often outperform more complex architectures in contexts with limited data and high interpretability demands. Logistic regression and random forest not only provided higher predictive performance but also allowed for more transparent model behaviour, facilitating insight into the relative influence of predictor variables—an essential consideration in psychological decision-making. In contrast, the opacity and instability of the artificial neural network underscore its limitations in domains where accountability, reproducibility, and interpretability are critical.

Despite the relatively large sample size (*N* = 4,155) for psychological research, it may still be insufficient for training feedforward neural networks with multiple layers and high parameter complexity. This limitation, combined with the relatively small number of features (*n* = 13), likely contributed to the models’ overfitting and instability. These findings are consistent with prior research indicating that neural network-based models require extensive data and careful regularization to avoid performance degradation (Geman et al., 1992; Hawkins, 2003). Feature selection strategies, such as dimensionality reduction or domain-driven index construction, may offer viable solutions in future studies aiming to enhance neural network-based model performance in psychological datasets.

An additional methodological contribution of the present study concerns the explicit treatment of noise. Variability in predictive performance across repeated training iterations was interpreted as indirect evidence of susceptibility to noise inherent in the data. Decision trees and neural networks proved particularly sensitive, whereas random forests showed greater robustness, consistent with their ensemble-based design. Treating noise as a central methodological dimension highlights that model evaluation in psychology must consider not only accuracy and generalization but also stability under random perturbations (Dietterich, 1995; Grandvalet and Bengio, 2005).

The broader implications of these results point to a continued role for traditional machine learning models in psychology, particularly for classification tasks involving structured data and moderate sample sizes. Logistic regression and random forest provide robust, interpretable, and computationally efficient tools, making them highly suitable for applied settings such as university admissions. Nonetheless, as the field continues to evolve, there remains a need to refine and adapt neural network-based model approaches to the specific challenges of psychological research - especially regarding small sample sizes, missing data, and the curse of dimensionality. Advances in regularization techniques, model compression, and domain-informed architecture design may help bridge this gap in future investigations.

Collectively, this study indicates that model selection in psychological data analysis must be guided not only by considerations of predictive accuracy but also by attention to overfitting risk, interpretability, the structural characteristics of the data, and robustness to noise. Traditional machine learning methods currently offer a more reliable and transparent approach for psychological applications, particularly when working with limited and structured datasets. Recent empirical applications of machine learning in psychological prediction contexts further support the robustness of ensemble methods such as random forests in structured datasets (Zhang et al., 2023).

Model performance was primarily reported using point estimates. Future research may benefit from uncertainty quantification through resampling procedures, confidence intervals, or statistical significance testing to further assess the robustness and practical relevance of observed performance differences.

The present findings should be interpreted considering several limitations. The empirical evaluation is based on a single university admissions dataset, which restricts the generalizability of the results. Observed performance differences may reflect characteristics specific to this dataset rather than general properties of psychological data. Replication across additional datasets and institutional contexts would be required to establish broader external validity. Nevertheless, the dataset represents a typical applied psychological selection context, supporting the relevance of the findings for comparable real-world settings.

## Data Availability

The dataset contains sensitive personal information collected as part of university admissions procedures. In accordance with the General Data Protection Regulation (GDPR) and institutional data protection policies, the raw data cannot be shared publicly. Only aggregated results and analysis scripts can be made available upon reasonable request. Requests to access these datasets should be directed to Marie-Luise Leitner, marie.leitner@uni-graz.at.
