# Correction: Comparing machine learning and artificial neural network models in psychological research: a ROC-based analysis

**DOI:** 10.3389/fpsyg.2026.1917351

**Published:** 2026-07-22

**Authors:** Marie-Luise Leitner, Martin Arendasy

**Affiliations:** Department of Psychology, University of Graz, Graz, Austria

**Keywords:** artificial neural network, decision tree, feature importance, logistic regression, machine learning, noise, overfitting, receiver operating characteristic (ROC)

The reference for Westin et al. (2002) was erroneously written as Westin, C. F., Maier, S. E., Mamata, H., Nabavi, A., Jolesz, F. A., and Kikinis, R. (2001). Processing and visualization for diffusion tensor MRI. *Medical Image Analysis, 6*(2), 93–108. doi: 10.1016/S1361-8415(01)00040-9.

It should be:

Westin, C.-F., Maier, S. E., Mamata, H., Nabavi, A., Jolesz, F. A., and Kikinis, R. (2002). Processing and visualization for diffusion tensor MRI. *Med. Image Anal*. 6, 93–108. doi: 10.1016/S1361-8415(02)00053-1

The reference for Zhang et al. (2017) was erroneously written as Zhang, C., Bengio, S., Hardt, M., Recht, B., and Vinyals, O. (2017). Understanding Artificial Intelligence requires rethinking generalization. *International Conference on Learning Representations (ICLR)*. https://arxiv.org/abs/1611.03530.

It should be:

Zhang, C., Bengio, S., Hardt, M., Recht, B., and Vinyals, O. (2017). Understanding deep learning requires rethinking generalization. *arXiv*. [preprint]. arXiv:1611.03530. doi: 10.48550/arXiv.1611.03530

The reference for Zhang et al. (2023) was erroneously written as Zhang, X., Zhao, Y., and Liu, H. (2023). Identifying the predictors of severe psychological distress using machine learning approaches: A random forest model comparison. *Journal of Affective Disorders Reports, 11*, 100485. doi: 10.1016/j.jadr.2023.100485.

It should be:

Zhang, X., Ren, H., Gao, L., Shia, B.-C., Chen, M.-C., Ye, L., et al. (2023). Identifying the predictors of severe psychological distress by auto-machine learning methods. *Inform. Med. Unlocked* 39:101258. doi: 10.1016/j.imu.2023.101258

The reference for Quinlan (1996) was erroneously written as Quinlan, J. R. (1996). Improved use of continuous attributes in C4.5. *J. Artif. Intell. Res*. 4, 77–90. doi: 10.1613/jair.279

It should be:

Quinlan, J. R. (1996). Improved use of continuous attributes in C4.5. *J. Artif. Intell. Res*. 4, 77–90.

The reference for Balcan & Sharma (2024) was erroneously written as Balcan, M. F., & Sharma, Y. (2024). Understanding robustness of decision trees. *Proceedings of the 41st International Conference on Machine Learning (ICML 2024)*. doi: 10.48550/arXiv.2402.12345.

It should be:

Balcan, M.-F., and Sharma, D. (2024). “Learning accurate and interpretable decision trees,” in *Proceedings of the fortieth conference on uncertainty in artificial intelligence*, Vol. 244 (PMLR), 288–307.

The original version of this article has been updated.

